# Work routines moderate the association between eveningness and poor psychological well-being

**DOI:** 10.1371/journal.pone.0195078

**Published:** 2018-04-06

**Authors:** Felipe Gutiérrez Carvalho, Camila Morelatto de Souza, Maria Paz Loayza Hidalgo

**Affiliations:** 1 Chronobiology and Sleep Laboratory, Hospital de Clínicas de Porto Alegre, Porto Alegre- RS, Brazil; 2 Psychiatry and Behavior Sciences Post-Graduation Program, Universidade Federal do Rio Grande do Sul, Porto Alegre- RS, Brazil; San Francisco Coordinating Center, UNITED STATES

## Abstract

Well-being is a useful screening method for the detection of mood disorders. Evidence associating psychological well-being with sleep-wake patterns exists, as well as associations with sleep-wake patterns, work-related parameters, and perceived self-efficacy. Despite the growing research regarding the relationship between these factors and mental health, there are few studies that analyze them together. OBJECTIVE: To investigate if the association between sleep-wake patterns and psychological well-being is mediated or moderated by perceived self-efficacy, work flexibility and work routines. MATERIAL AND METHODS: This cohort study was performed in southern Brazil. A sample of 987 individuals was analyzed (66.9% women; mean age = 43.9 years). Work routines parameters and work schedule flexibility were evaluated, most participants were farmers (46%) and most worked 7 days a week (69.1%). Munich Chronotype Questionnaire (MCTQ) was administered for evaluation of sleep-wake patterns, General Self-Efficacy Scale (GSE) for assessment the participants’ beliefs about how they coped with daily hassles, and World Health Organization Five-item Well-being Index (WHO-5) for evaluation of psychological well-being levels. Moderation and mediation models were tested. RESULTS: The moderation model showed influences of work end time on the relationship between sleep onset time and psychological well-being (R^2^ = 0.147; F = 24.16; p<0.001). The final regression model showed an association of psychological well-being with sex (Beta = -0.086; p = 0.004), sleep onset time (Beta = -0.086; p = 0.006), and self-efficacy (Beta = 0.316; p<0.001); the work end time showed association in the interaction with sleep onset time (Beta = -0.075; p = 0.016). CONCLUSION: The findings support the direct association of psychological well-being with sleep-wake patterns and self-efficacy, and show an interaction between work routines and sleep-wake patterns. Our results draw attention to the importance of the interplay between individual and social rhythms in relation to psychological well-being.

## Introduction

Psychological well-being is an important measure of general aspects of health [1], and a useful screening method to detect mood disorders [2]. Several factors may influence this outcome. There is evidence associating psychological well-being with sleep-wake patterns [3, 4]. This relationship may be mediated by other factors associated to psychological well-being, including stress response abilities [5, 6], psychosocial working conditions [7–9] and work routines [10]. Moreover, the circadian disruption observed in the extreme interplay between sleep-wake patterns and work schedules, such as shift work, are associated to poorer health outcomes [11–13].

The individual circadian rhythmic expression, defined as the circadian typology, consists of different patterns of synchronization between endogenous and exogenous temporal factors. Sleep and wake time patterns have been shown to have a normal distribution in the population [14]. The pattern of circadian rhythm synchronization refers not only to sleep-wake cycles but also to an individual’s adjustment to a variety of physical and mental activities [15, 16]. The chronotypology also reflects emotional adaptiveness and affective stability profiles [17]. There is evidence that individual circadian rhythmic expression affects our biological and psychological functioning, several studies have shown the association between individual circadian patterns and health-related outcomes [11, 18]. Regarding chronotypology and mental health, most of the evidence shows an association between late chronotypes and poorer outcomes, such as depressive symptoms [19–21], mood disorders [22], and maladaptive substance use [23].

The circadian rhythm patterns play an important role in individual physiology, and misalignment between social and physiological demands may result in chronodisruption. Differences may vary from extreme changes, such as shift work schedules, to slight variations. Even the difference in rhythms during workdays and days off, a phenomenon which is called “social jet lag”, is associated to worse mental health outcomes [20, 24]. Although the pathways in this process are not yet fully understood, chonodisruption may lead to deregulation of the Hypothalamic-pituitary-adrenal axis, one of the main stress response endogenous pathways [25]. Therefore, the circadian rhythm adaptation may also be related to the ways in which people cope to stressful events [26]. The intrapsychic and action-oriented processes of attempting to manage the demands created by stressful events appraised as taxing or exceeding our resources are defined as Coping [27]. There are studies analyzing the pathways between coping abilities and psychological well-being [5]. The coping process involves individual resources such as optimism, perceived self-efficacy, self-esteem and social support [28]. The perceived self-efficacy is understood as the individual’s beliefs in his or her own ability to cope with stressful demands, and affects various aspects of human functioning through four processes: cognitive, motivational, affective, and environmental selection [29, 30]. Association and moderation/mediation studies suggest that self-efficacy is related to sleep-wake patterns [31, 32], and to well-being in general health morbidity [6].

Although there is consistent evidence of the importance of all these factors in relation to health, no studies investigating the interrelationship among psychological well-being, circadian typology patterns, self-efficacy and work-related parameters were found. In addition, there is evidence suggesting that the relationship between sleep-wake patterns and well-being may be mediated by other factors [33]. There are methodological strategies which promote a better understanding of the pathways among different factors, as the Mediation and Moderation analyses [34]. This methodological approach may shed light on how and when the factors associate to the outcome. The mediation analysis compares direct and indirect effects between the outcome and the factors. It promotes a better understanding of how a factor (X) behaves towards an outcome (Y) through another factor (Mediator) and clarifies causal inferences in the hypothesis. The moderation analysis includes the interaction variables in the equations. It promotes a better understanding of when the association happens, as it examines the possible different effects of a factor X on an outcome Y in relation to another factor (in other words, the interaction between X and Moderator). Both the analyses are important as they show details of the interrelationship among the elements in evaluation which may not be seen in a regular analysis.

### Aims of the study

**Fig 1:**
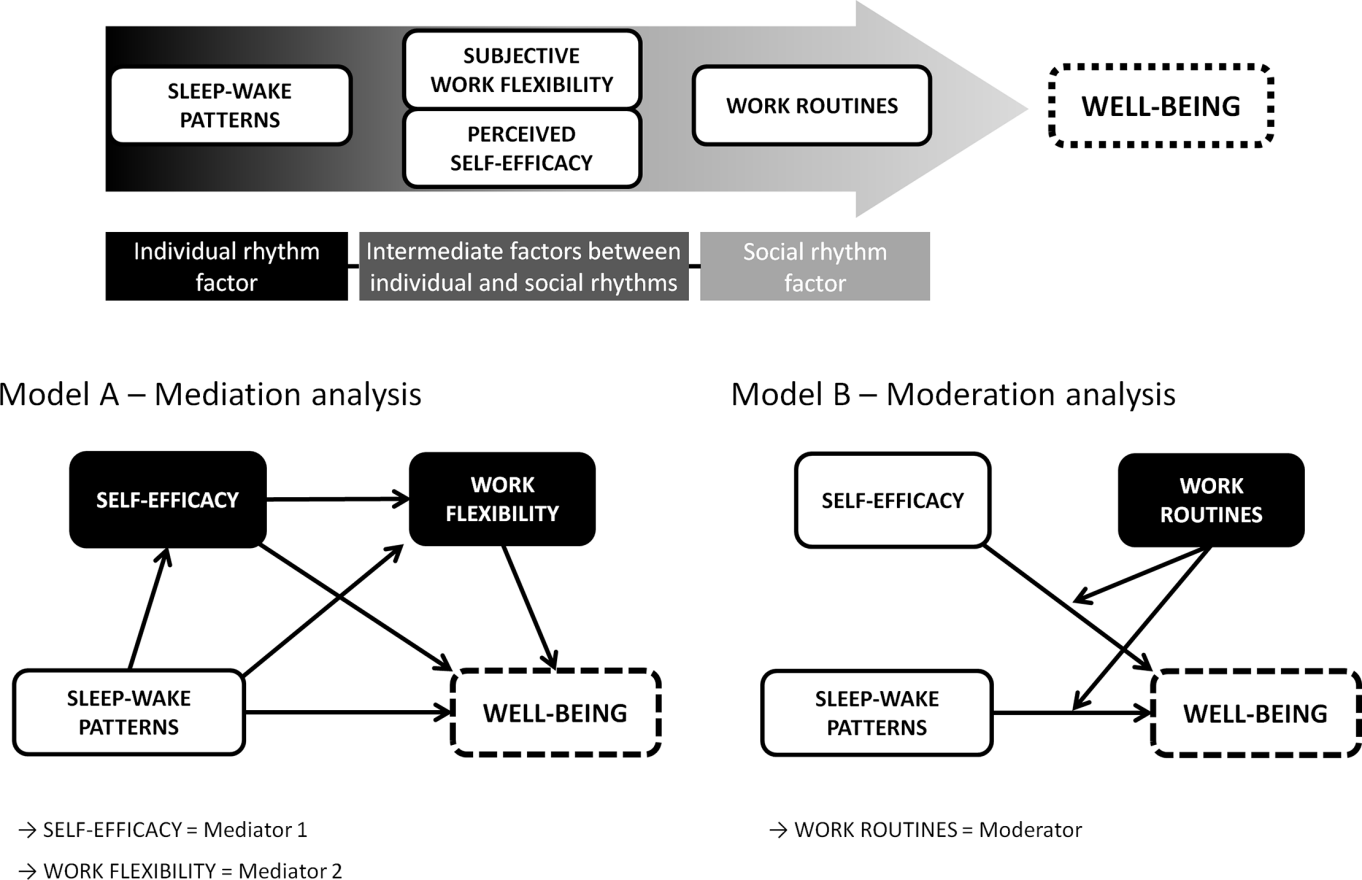
Hypothesis and tested path models. Model A. Simple and serial multiple mediation effects of self-efficacy and perceived work schedule flexibility on the relationship between sleep-wake patterns and psychological well-being; Model B. Moderation effects of work routines on the relationship of psychological well-being with sleep-wake patterns and perceived self-efficacy.

The aim of the present study was to analyze if the association between sleep-wake patterns and psychological well-being is mediated by stress-adaptive abilities, and moderated by social demands. A mediation model of perceived self-efficacy and work flexibility, and a moderation model of work routine parameters were used. The hypothesized models are shown in Fig 1.

## Materials and methods

### Participants

The present data was collected as part of an epidemiological survey performed in a rural area (Vale do Taquari) in southern Brazil, where most of the population is of German and Italian descent [35]. From an initial sample of 6,506 participants, a block randomization was performed which included the same proportion of subjects for early, intermediate, and late chronotypes, in order to have a bigger number of individuals with more extreme chronotypes, so that extreme and intermediate groups could be properly compared. From this, 1,127 subjects were successfully re-interviewed (loss of 173 subjects). Participants without a work routine (n = 61) and with missing data in any of the questionnaires used were excluded (n = 140). The final sample consisted of 987 subjects (66.9% female; mean age of 43.9 ± 12.51).

Health status and substance use were assessed. Presence of disease was self-reported by 345 participants (35%), and current use of medication was reported by 369 people (37.4%). Alcohol consumption was reported by 514 participants (52.1%), current tobacco use was reported by 103 participants (10.4%), and past tobacco use was reported by 95 participants (9.6%). Regarding to occupational data, 454 (46%) were farmers, 317 (32.1%) worked with unskilled or semi-skilled services, 60 (6.1%) were manufacturing workers, 38 (3.9%) were retail workers, 26 (2.6%) worked with skilled services, and 13 (1.3%) were students. It should be noted that 77 subjects (7.8%) were formally retired at the moment of the interview and 2 subjects (0.2%) considered themselves unemployed, but all of them reported regular work routines. Regarding working days/week, 305 (30.9%) worked from 1 to 6 days/week, and 682 (69.1%) worked 7 days/week. The literature suggests a good correlation between working-day and free-day sleep-wake patterns [10]. Since the sample was composed mostly of 7-day a week workers, the data on working days were used for analysis. A comparison of the chronotype distribution (midpoint of sleep on working days) between this study sample and the general sample evaluated in the first phase showed higher prevalence of earlier circadian typology in this sample (S1 Fig, available as supporting information). The characteristics of the sample are shown in Table 1.

**Table 1:** Characteristics of the sample (N = 987).

		n (%) or mean (±SD)
Sex		
	Male	327 (33.1%)
	Female	660 (66.9%)
Age		43.93 (±12.51)
Sleep-wake patterns on working days		
	Sleep onset time	10:23pm (±1h19min)
	Sleep end time	6:09am (±1h01min)
	Sleep duration	7h46min (±1h15min)
	Midpoint	2:16am (±1h)
Self-efficacy score		34.59 (±4.34)
Work schedule flexibility		
	Very flexible	484 (49.0%)
	Flexible	383 (38.8%)
	Inflexible	84 (8,5%)
	Very Inflexible	36 (3.6%)
Work routines		
	Work start time	7:21am (±1h43min)
	Work end time	5:52pm (±2h19min)
	Work duration	10h32min (±2h48min)
	Midpoint	12:36pm (±1h29min)

### Study design and measurements

In this cohort study, participants were evaluated in their homes by trained interviewers on two occasions. At the first assessment, interviewers administered the Munich Chronotype Questionnaire (MCTQ) to evaluate sleep-wake patterns and collected demographic data, working characteristics, and health-related data. One year later, the World Health Organization Five-item Well-being Index (WHO-5) was administered for evaluation of psychological well-being levels, the General Self-Efficacy Scale (GSE) was administered to assess the participants’ beliefs about how they coped with daily hassles, and the work schedule flexibility was evaluated. The mean interval between the two assessments was 13.80 months (±5.86). The interval between the initial and the final assessments was planned to be approximately 12 months, in order to minimize possible confounding factors related to seasonal variations–similar light and dark periods during both the assessments. In order to permit causal inferences, the variables evaluated in the first and in the second visit were chosen due to the statistical procedure involved in the mediation analysis–evaluation of factor and cofactors in the first visit, and of outcome and mediators in the second.

#### Measurement of sleep-wake patterns

The MCTQ is a self-administered, structured questionnaire used to collect information on sleep-wake habits and sunlight exposure on free and working days separately. The following information was obtained regarding sleeping patterns: bedtime, sleep latency, sleep duration, wake-up time, fully awake time. The chronotype was considered according to the midpoint of sleep, which is calculated by dividing the sleep duration by two and adding the resulting number to the sleep onset time. Although the MCTQ assesses actual sleep times separately for work and work-free days, work-free day variables were not considered, since the sample was composed mainly of subjects working 7 days/week. A Brazilian-Portuguese version of the MCTQ was used (http://www.euclock.org/).

#### Self-efficacy measurement

The GSE, a self-answered structured questionnaire was used to assess general sense of perceived self-efficacy to cope with daily hassles as well as adaptation after experiencing all kinds of stressful life events. A validated Brazilian Portuguese version was used [36].

#### Measurement of work schedule flexibility

A simple question to gauge perceived work schedule flexibility was used. The answer was rated on a four-point scale–“very flexible,” “flexible,” “inflexible,” or “very inflexible.” As the answer suffers the influence of subjective considerations, it was not used as an objective measure of work schedule flexibility, but as an individual perception of working demands. Therefore, it should be pointed that both General Self-Efficacy and Work Schedule Flexibility were not considered mainly individual or social rhythm factors, but intermediate factors which may reflect the interplay between the responder and his or her social demands.

#### Measurement of work routine parameters

Working day start and end time, work duration, and number of working days per week were used as measures of work routines. These data originated from the sociodemographic questionnaire, which included questions about the occupation.

#### Measurement of psychological well-being

The WHO-5 assesses psychological well-being patterns with five simple questions. This instrument takes into consideration the previous 2 weeks. The score is rated on a six-point Likert scale, from 0 to 5, which generates a raw score ranging from 0 to 25. Originally, a total score of less than 13 points indicates poorer well-being, and further clinical investigation for depression should be considered [37]. However, a Brazilian Portuguese validation study suggests a cut-off of <19/20, with higher sensitivity [38].

### Ethical aspects

The experimental protocol followed international ethical standards [39]. The Ethics Committee at Hospital de Clínicas de Porto Alegre approved the study protocol (Project 08–087 GPPG/HCPA, CONEP 15155) and all participants gave written informed consent.

### Statistical analysis

All data were entered into the IBM Statistical Package for the Social Sciences (SPSS) 18. Sample demographics were expressed as means ± standard deviation (SD) or number of cases (n) and percentages. We defined normality through skewness and kurtosis of ± 1, and this was considered for the choice of the appropriate test for each variable distribution. Since the WHO-5 score and the GSE score presented skewness and kurtosis values out of the expected for normal distribution, the values were square-transformed for analysis. The square-transformed variables revealed acceptable values of skewness and kurtosis: WHO-5 score, Sk = -0.074, K = -0.670, and GSE score, Sk = -0.649, K = -0.211.

Pearson’s test was used to compare two continuous variables; Student’s t test was used for comparisons between two groups; analysis of variance (ANOVA)/Post-hoc Tukey were used for comparison of variables with three or more groups. Furthermore, to assess possible confounding effects and collinearity of variables, a multivariate linear hierarchical regression analysis with the WHO-5 score was used as the dependent variable. This analysis only included variables which presented a correlation on the univariate analysis with a statistical difference of at least p ≤ 0.2.

The multivariate linear hierarchical regression analysis was composed of three models: the first model included age, sex, and sleep onset time; the second model added self-efficacy score and work schedule flexibility; and the third model added work end time. The models followed a progressive inclusion from individual to social rhythm factors, with the inclusion of individual characteristics in Model 1, intermediate characteristics in Model 2, and, finally, social rhythm characteristics in Model 3 (as illustrated in Fig 1). The sleep onset time and the work end time correspond to the sleep-wake patterns and the work related parameters representative variables respectively and were chosen due to the better correlation to psychological well-being (sleep onset time: r = -0.14, p<0.001; work end time: r = -0.06, p = 0.062).

Moderation and mediation analyses were performed in order to clarify when and how individual and social rhythm characteristics are associated to the psychological well-being. This methodological approach aims to promote a better understanding of the interrelationship between outcome and the factors in evaluation. The procedure used for these analyses attended the standard recommendations [40, 41]. For the moderation analysis, a 4^th^ model was included into the regression, which adds the interaction between sleep-wake patterns and work routine parameters. For all analyses, a two-tailed p < 0.05 was considered statistically significant. Mediation and moderation analyses were carried out using PROCESS, which is a computational macro available online for SPSS [41]. All the continuous variables were z-standardized [42]. PROCESS uses bias-corrected bootstrapping to generate confidence intervals [43]. This procedure addresses the problem of bias resulting from the asymmetric and non-normal sampling distributions of an indirect effect [44]. One thousand bootstrap resamples were used to generate bias-corrected 95% confidence intervals for the indirect effect. In the simple mediation models, we also performed the Sobel test, which is an alternative to test the indirect effect in terms of power and intuitive appeal [45].

## Results

Table 2 summarizes the results of the univariate analysis for WHO-5 score, showing the most relevant characteristics for the inclusion to multivariate analysis. Due to their stronger correlation with psychological well-being, sleep onset time and working end time were used as the main variables reflecting sleep-wake patterns and work routine parameters. Poorer psychological well-being is associated with later sleep onset and with poorer self-efficacy, and shows a trend with work end time (S2 and S3 Figs, available as supporting information). ANOVA was performed to compare the various occupations and the groups with a different number of working days during the week, showing statistical differences which were not maintained after post-hoc analysis.

**Table 2:** Univariate analysis for the association between the studied variables and psychological well-being scores (N = 987).

		Psychological well-being score (mean ±SD)	Test	P-value
**Sex**			**t = 4.11**	**<0.001**
	Male	19.15 (±4.26)		
	Female	17.87 (±4.73)		
**Age**			**r = 0.11**	**0.001**
Sleep-wake patterns on working days				
	Sleep onset time*		**r = -0.14**	**<0.001**
	**Sleep end time**		**r = -0.06**	**0.045**
	**Sleep duration**		**r = 0.10**	**0.002**
	**Midpoint**		**r = -0.13**	**<0.001**
**Self-efficacy score**			**r = 0.33**	**<0.001**
Work schedule flexibility			F = 2.58	0.052
	Very flexible	18.58 (±4.53)		
	Flexible	18.24 (±4.63)		
	Inflexible	17.23 (±5.11)		
	Very Inflexible	17.44 (±4.11)		
Work routines				
	Work start time		r<0.01	0.877
	Work end time*		r = -0.06	0.062
	Work duration		r = -0.05	0.099
	Midpoint		r = -0.04	0.177

*Sleep onset time and Work end time showed a better correlation with Psychological well-being in comparison to the other variables related to Sleep-wake patterns on working days and to Work routines respectively.

The mediation analysis showed no statistically significant effect of self-efficacy and working schedule flexibility on the relationship between sleep onset time and psychological well-being. Three different models for this analysis were used: one for the mediation effect of self-efficacy individually, one for the mediation effect of working schedule flexibility individually, and one including the mediation effects of both factors (serial multiple mediation model, Fig 1 - Model A). Age and sex were included as covariates in all the models, working schedule flexibility was included as a covariate in the first model, and self-efficacy was included as a covariate in the second model. For further information about the statistics used in this analysis, S1 and S2 Tables are available as supporting information.

Models 1 to 4 in Table 3 show the hierarchical regression procedure performed to test Moderation effect (Fig 1 - Model B). Models 1 to 3 show a hierarchical progression, from individual to social rhythm factors; Model 4 shows the moderation effect of work end time on the relationship between sleep onset time and psychological well-being. Moderation analysis was also performed to assess the relationship between self-efficacy and psychological well-being (R^2^ = 0.142; F = 23.19; p<0.001), but the interaction in this model was not statistically significant (p = 0.882). In both analyses, the interactions among all the covariates were controlled, and there were no other significant interactions.

**Table 3:** Summary of hierarchical regression analysis for variables and interactions predicting psychological well-being score (n = 987).

	**Model 1**				**Model 2**			
	B (SE)	Beta	Test	P-value	B (SE)	Beta	Test	P-value
Age	**0.067 (0.032)**	**0.067**	**2.061**	**0.040**	0.052 (0.031)	0.052	1.649	0.100
Sex	**-0.230 (0.067)**	**-0.108**	**-3.419**	**0.001**	**-0.168 (0.064)**	**-0.079**	**-2.608**	**0.009**
Sleep onset time	**-0.114 (0.033)**	**-0.114**	**-3.507**	**<0.001**	**-0.100 (0.031)**	**-0.100**	**-3.216**	**0.001**
Self-efficacy					**0.316 (0.030)**	**0.316**	**10.497**	**<0.001**
Work schedule flexibility					-0.032 (0.040)	-0.025	-0.811	0.417
Work end time								
Interaction 1								
	df = 3; 983				df = 5; 981			
	F = 12.86*				F = 31.56*			
	R^2^ = 0.038				R^2^ = 0.139			
	**Model 3**				**Model 4**			
	B (SE)	Beta	Test	P-value	B (SE)	Beta	Test	P-value
Age	0.052 (0.031)	0.052	1.643	0.101	0.052 (0.031)	0.052	1.659	0.098
Sex	**-0.180 (0.065)**	**-0.085**	**-2.794**	**0.005**	**-0.184 (0.064)**	**-0.086**	**-2.850**	**0.004**
Sleep onset time	**-0.093 (0.031)**	**-0.093**	**-2.978**	**0.003**	**-0.086 (0.031)**	**-0.086**	**-2.763**	**0.006**
Self-efficacy	**0.316 (0.030)**	**0.316**	**10.517**	**<0.001**	**0.316 (0.030)**	**0.316**	**10.536**	**<0.001**
Work schedule flexibility	-0.036 (0.039)	-0.028	-0.913	0.361	-0.036 (0.039)	-0.028	-0.906	0.365
Work end time	**-0.061 (0.030)**	**-0.061**	**-2.047**	**0.041**	-0.039 (0.031)	-0.039	-1.241	0.215
Interaction 1					**-0.075 (0.031)**	**-0.075**	**-2.410**	**0.016**
	df = 6; 980				df = 7; 979			
	F = 27.08*				F = 24.16*			
	R^2^ = 0.142				R^2^ = 0.147			

* P-value <0.001.

Model 1 = Individual rhythm factors.

Model 2 = Model 1 + intermediate factors between individual and social rhythms.

Model 3 = Model 2 + social rhythm factors.

Model 4 = Moderation analysis of the interaction between individual (sleep onset time) and social (work end time) rhythm factors.

Interaction 1 = Sleep onset time*work end time.

**Fig 2:**
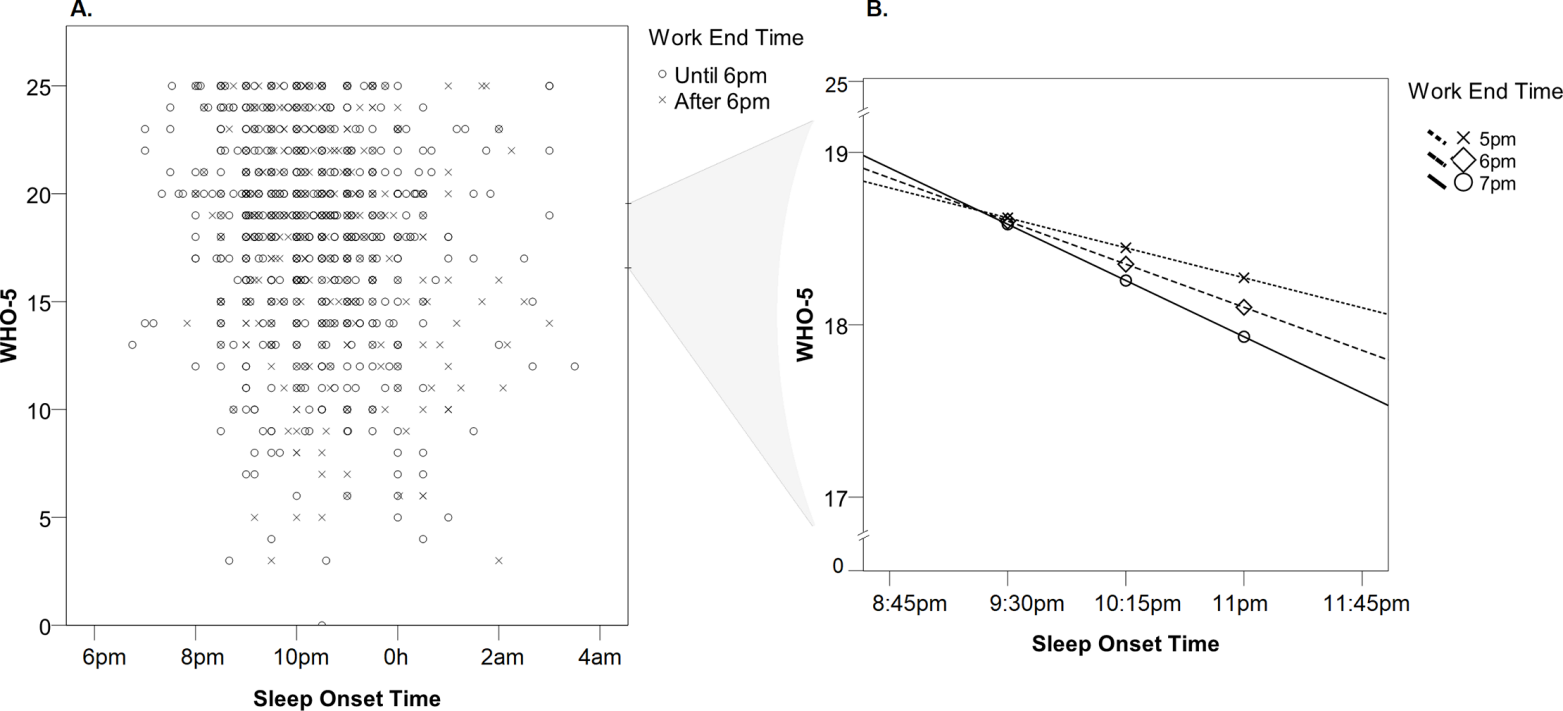
Interrelationship between WHO-5, sleep onset time, and work end time. A. Scatterplot graph with the total distribution of WHO-5 scores according to sleep onset time for categorical work end time—until and after 6pm (cutoff: median value); B. Conditional effect of sleep onset time on psychological well-being at the P25, P50 and P75 values of work end time.

**Fig 3:**
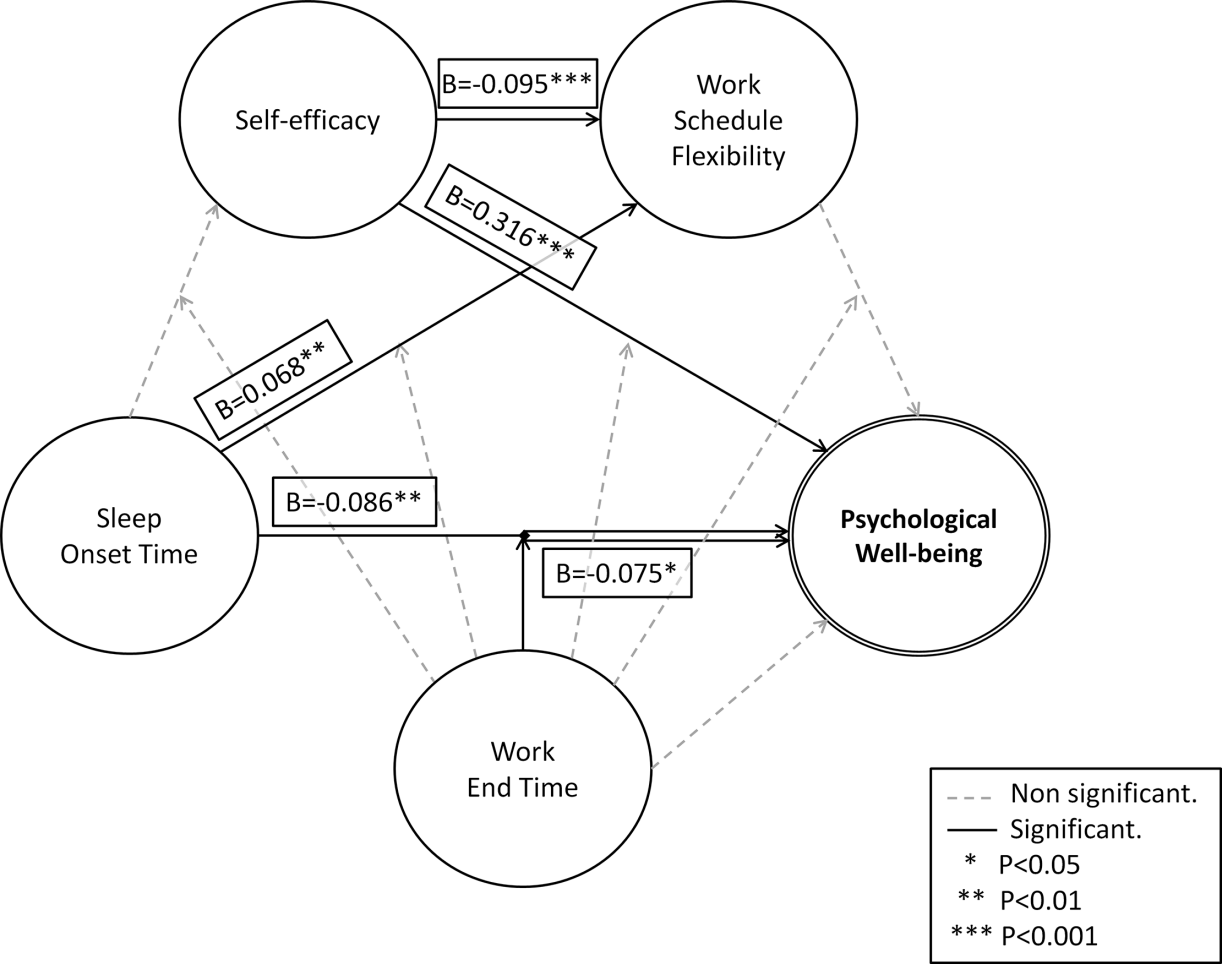
Final statistical diagram.

Fig 2 illustrates the interaction between sleep onset time and work end time in relation to psychological well-being. Fig 2A shows the total distribution of psychological well-being scores according to sleep onset time, divided between work end time until and after 6 pm (median value). In Fig 2B, the moderation effect is illustrated in different values of the work end time– 5 pm, 6 pm and 7 pm, which are the percentiles 25, 50 and 75 respectively. Table 4 shows the equations of the conditional effect of sleep onset time on psychological well-being at the values of the work end time used in Fig 2B—for better graphic illustration, the values used in this analysis correspond to nonz-standardized independent and dependent variables. Fig 3 shows the final statistical diagram.

**Table 4:** Conditional effect of sleep onset time on Psychological well-being at values of the work end time.

Work End Time	B*	SE	t	p	LLCI	ULCI
5pm	-0.232	0.125	-1.851	0.064	-0.478	0.014
**6pm**	**-0.333**	**0.118**	**-2.831**	**0.005**	**-0.565**	**-0.102**
**7pm**	**-0.435**	**0.128**	**-3.398**	**0.001**	**-0.686**	**-0.184**

*Values of B for non z-standardized independent and dependent variables.

## Discussion

Prior to this study, literature evaluating the relationship between psychological well-being with sleep-wake patterns, self-efficacy, and work routine parameters considering individual and social rhythms was not found. Our main findings suggest (i) sleep-wake patterns are associated to psychological well-being, and this association seems to interact with work routines; (ii) self-efficacy scores are highly associated to psychological well-being, and seem to have no interactions with factors related to work; and (iii) work routine parameters seem to have no independent association, but may exert a moderation effect on the relationship between sleep-wake patterns and psychological well-being.

Regarding the initial hypotheses, two different models were tested in order to have a better understanding regarding the impact of each of these factors on the association between psychological well-being and sleep-wake patterns. Models 1 to 3 of the hierarchical linear regression showed direct associations of sleep-wake patterns and self-efficacy in relation to well-being. Results regarding sleep-wake patterns are similar to those reported by previous studies [20, 22, 24], showing poorer psychological well-being in later chronotypes. An important characteristic of the present sample, however, is the earlier chronotype as compared to other populations [14], which may be related to several factors, such as genetic characteristics, occupational adaptation (farming is a major economic activity in this region, and individuals who do not adapt to early morning activities may prefer to live elsewhere), or even influenced by the age of the participants (the mean ages between the initial and the second phase samples were 42 *vs* 43.9 years). Our findings regarding the direct association between self-efficacy and psychological well-being were also similar to those previously reported [6].

The Model 4 of the hierarchical regression corresponds to the moderation analysis (Fig 1, Model B), and was successfully tested, showing a moderation effect of work routine parameters on the relationship between sleep-wake patterns and psychological well-being. After the inclusion of the interaction variable, the significance of work end time was lost, showing that this variable is dependent on sleep onset time. This may be considered an important example of individual and social rhythm interplay, and an interesting finding regarding individual characteristics and working conditions. In the population studied, the economic activity greatly influences working parameters. The main occupation of participants is farming, which is of special interest because of the strong relationship between this occupation and diurnal activities. The adaptation of late chronotypes to this activity is expected to be more difficult, making this a favorable scenario to study self-efficacy and psychological well-being. One of the difficulties to compare this parameter is that most of the data in the literature evaluate shift work, and do not evaluate diurnal rhythmic work routines as was done in the present study. This hinders the comparison with earlier studies. Our analysis of the relationship between psychological well-being and work parameters showed a trend of worse outcomes related to later work end times, and these results are similar to the results of previous studies [11–13]. Interestingly, sleep onset time had a stronger correlation with psychological well-being when compared to the other sleep-wake patterns, probably because this variable is more independent in relation to work routines, and may be the best parameter to infer individual chronotype. In turn, the sleep end time presented a very poor association with psychological well-being. This might be explained by the need to adjust sleep end time to the demands of the work schedule, which could result in an “artificial” waking time. In urban populations, the light pollution during the dark cycle and the massive manipulation of the waking time promoted by the use of alarm clocks may amplify this effect, and a deeper misalignment between individual and social demands is expected.

The mediation analysis (Fig 1, Model A) was not successfully replicated since indirect effects of self-efficacy and perceived work schedule flexibility on the relationship between chronotype and psychological well-being were not found. Even considering the relationship between chronodisruption and stress response [25], studies analyzing the mediation effect of self-efficacy on the relationship between chronotype and psychological well-being were not found as well. One study with structural equation modeling of coping abilities, distress, and psychological well-being has found indirect effect among these factors, but sleep-wake patterns were not included in the analysis [5]. Nevertheless, there is evidence suggesting indirect psychiatric components on this relationship, such as maladaptive substance use [33]. Some studies have detected a mediation effect of sleep disturbance on the relationship between general self-efficacy and well-being [32], as well as mediation and multiple associations among insomnia symptoms, sleep-related dysfunctional cognitions, general self-efficacy, and well-being [31]. These studies considered sleep-wake patterns as mediation factors for a correlation between self-efficacy and well-being. Regarding the perceived working schedule flexibility measure, specific studies regarding this issue were not found in the literature. Most of the studies have considered multiple work routine issues and their relationship with social and familial commitments, although it remains unclear if these may be considered objective measures, or if they suffer influence of subjective perceptions [7]. More studies are needed to clarify which factors may be involved in these relationships.

The dependence of work routine parameters on sleep-wake patterns regarding psychological well-being is an important finding that requires evaluation. Moreover, our findings also suggest a strong independent association of chronotype and self-efficacy on psychological well-being. These are remarkable findings which show the importance of these factors for general mental health concerns. Therefore, more investigation is needed to understand the possible pathways that may influence and be influenced by circadian rhythms, perceived self-efficacy, and working routines.

### Strengths and limitations

Causal inferences are limited due to the results of mediation analysis. In relation to chronotype synchronization, a predominantly rural sample may behave very differently from an urban population. However, the strong influence of artificial light on the synchronization of biological rhythms could provide results with relevant confounding factors in a predominantly urban sample. Thus, in order to evaluate the sleep-wake pattern as the main individual rhythm factor, lower levels of artificial light contributed to clearer results regarding this biological background. The limited variation in working days per week may have been a limitation since most participants worked 7 days per week. However, there is evidence of a good correlation between working days and free days in terms of sleep-wake patterns [10]. In our sample, the correlation between working and free-day sleep onset time was confirmed (Pearson’s r = 0.661; p<0.001).

## Conclusions

In sum, the final model shows that lower psychological well-being scores correlate with female gender, later sleep onset times, and lower self-efficacy. In addition, an interaction was detected between work end time and sleep onset time, showing worse outcomes among late chronotypes with late work routines than with early work routines. Our results support most of the findings in the literature and show new insights regarding the importance of the interplay between individual and social rhythms in relation to psychological well-being.

## Supporting information

S1 Fig Chronotype distribution: General sample vs. study sample. (TIF)

S2 Fig Sleep onset time and work end time according to WHO-5 score. Distribution of sleep onset time (A) and work end time (B) according to WHO-5 score.
(TIF)

S3 Fig Relationship between WHO-5 and GSE scores. (TIF)

S1 Table Mediation analysis. Regression analysis predicting a mediation effect of self-efficacy and working schedule flexibility on sleep onset time and well-being status.
(PDF)

S2 Table Mediation analysis. Direct, indirect, and total effects of sleep onset time on psychological well-being.
(PDF)
