# Supplementary figures and images for: Work routines moderate the association between eveningness and poor psychological well-being
Source: PLoS One. 2018 Apr 6;13(4):e0195078. doi: 10.1371/journal.pone.0195078 (PMC5889056; doi:10.1371/journal.pone.0195078)

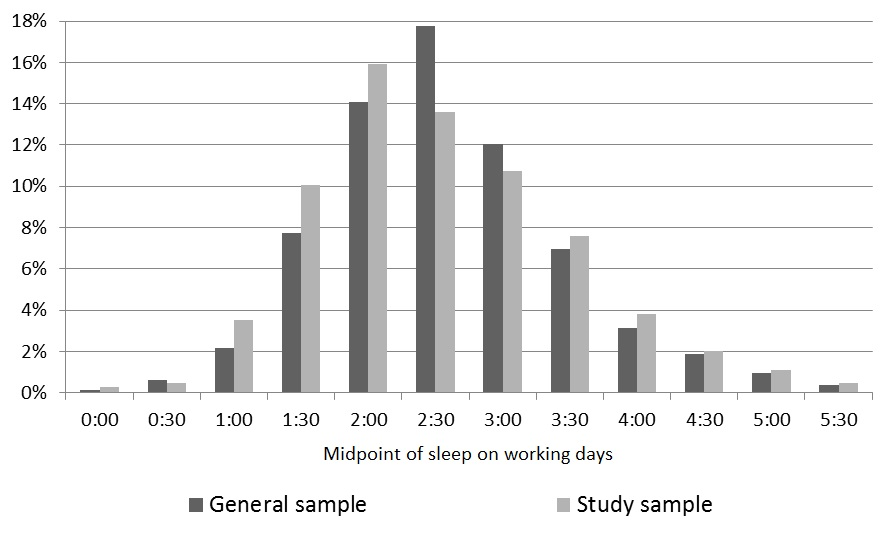

Supplement: S1 Fig — (TIF) [file pone.0195078.s001.tif]

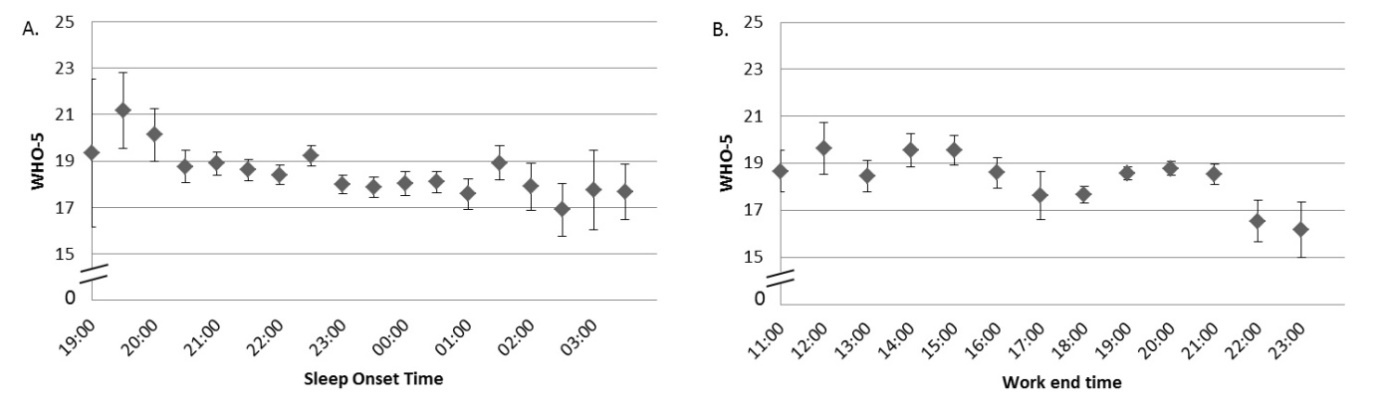

Supplement: S2 Fig — Distribution of sleep onset time (A) and work end time (B) according to WHO-5 score. (TIF) [file pone.0195078.s002.tif]

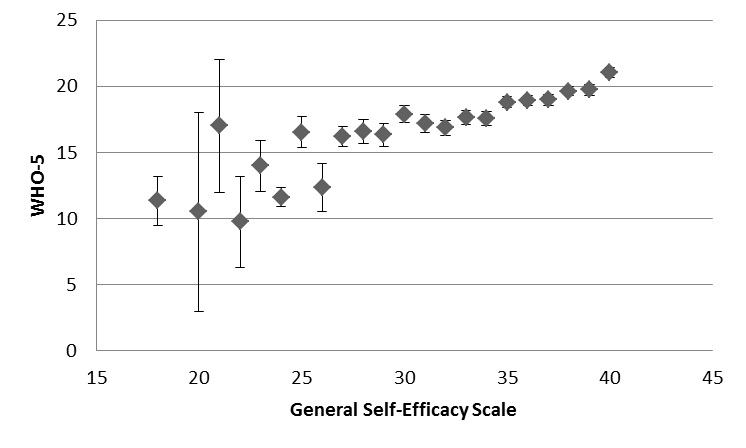

Supplement: S3 Fig — (TIF) [file pone.0195078.s003.tif]
