# Supplementary material for: Work routines moderate the association between eveningness and poor psychological well-being
Source: PLoS One. 2018 Apr 6;13(4):e0195078. doi: 10.1371/journal.pone.0195078 (PMC5889056; doi:10.1371/journal.pone.0195078)
Supplement: S1 Table — Regression analysis predicting a mediation effect of self-efficacy and working schedule flexibility on sleep onset time and well-being status. (PDF) [file pone.0195078.s004.pdf]

Table S1 - Regression analysis predicting a mediation effect of self-efficacy and working schedule flexibility on sleep onset time and well-being status.

	Outcome	Predictor	Mediator	B	SE	t	95% CIs		P-value
Model 1									
	WHO-5	Sleep Onset Time	Self-efficacy	-6.07	7.02	-0.86	-19.86	7.71	0.387
Model 2									
	WHO-5	Sleep Onset Time	Working Schedule Flexibility	0.05	0.02	2.54	0.01	0.08	0.011
Model 3									
	WHO-5	Sleep Onset Time	Self-efficacy	-8.44	7.05	-1.19	-22.55	5.66	0.231
			Working Schedule Flexibility	0.05	0.02	2.54	0.01	0.09	0.011

Models 1 and 2: Simple mediation effect on sleep onset time and well-being status.
Model 3: Serial multiple mediation effect on sleep onset time and well-being status.
