# Supplementary material for: Work routines moderate the association between eveningness and poor psychological well-being
Source: PLoS One. 2018 Apr 6;13(4):e0195078. doi: 10.1371/journal.pone.0195078 (PMC5889056; doi:10.1371/journal.pone.0195078)
Supplement: S2 Table — Direct, indirect, and total effects of sleep onset time on psychological well-being. (PDF) [file pone.0195078.s005.pdf]

Table S2 - Direct, indirect, and total effects of sleep onset time on psychological well-being.

	B	SE	t	95% CI		P-value	Sobel test	
							Z	P-value
Model 1								
Total effect	-12.78	3.84	-3.33	-20.32	-5.24	<0.001	-	-
Direct effect	-11.72	3.65	-3.22	-18.88	-4.57	0.001	-	-
Indirect effect	-1.05	1.32	-	-3.88	1.51	-	-0.86	0.390
Model 2								
Total effect	-11.97	3.63	-3.29	-19.10	-4.83	0.001	-	-
Direct effect	-11.72	3.65	-3.22	-18.88	-4.57	0.001	-	-
Indirect effect	-0.24	0.33	-	-1.24	0.22	-	-0.72	0.469
Model 3								
Total effect	-13.45	3.83	-3.51	-20.97	-5.92	<0.001	-	-
Direct effect	-11.73	3.64	-3.22	-18.88	-4.57	0.001	-	-
Total Indirect effect	-1.72	1.36	-	-4.83	0.74	-	-	-
Indirect effect 1	-1.46	1.32	-	-4.63	0.69	-	-	-
Indirect effect 2	-0.01	0.02	-	-0.12	0.01	-	-	-
Indirect effect 3	-0.24	0.34	-	-1.12	0.25	-	-	-

Model 1: Simple mediation model. Mediator = Self-efficacy.
Model 2: Simple mediation model. Mediator = Working schedule flexibility.
Model 3: Serial multiple mediation model. Mediator 1 = Self-efficacy; Mediator 2 = Working schedule flexibility.
Indirect effect 1: Sleep onset time > self-efficacy > psychological well-being.
Indirect effect 2: Sleep onset time > self-efficacy > work schedule flexibility > psychological well-being.
Indirect effect 3: Sleep onset time > working schedule flexibility > psychological well-being.
